# Robust Detection, Segmentation, and Metrology of High Bandwidth Memory 3D Scans Using an Improved Semi-Supervised Deep Learning Approach

**DOI:** 10.3390/s23125470

**Published:** 2023-06-09

**Authors:** Jie Wang, Richard Chang, Ziyuan Zhao, Ramanpreet Singh Pahwa

**Affiliations:** Institute for Infocomm Research (I2R), Agency for Science, Technology and Research (A*STAR), 1 Fusionopolis Way, #21-01, Connexis South Tower, Singapore 138632, Singapore; wang_jie@i2r.a-star.edu.sg (J.W.); zhao_ziyuan@i2r.a-star.edu.sg (Z.Z.); ramanpreet_pahwa@i2r.a-star.edu.sg (R.S.P.)

**Keywords:** 3D semi-supervised Learning, 3D object detection, 3D semantic segmentation, contrastive learning, 3D metrology

## Abstract

Recent advancements in 3D deep learning have led to significant progress in improving accuracy and reducing processing time, with applications spanning various domains such as medical imaging, robotics, and autonomous vehicle navigation for identifying and segmenting different structures. In this study, we employ the latest developments in 3D semi-supervised learning to create cutting-edge models for the 3D object detection and segmentation of buried structures in high-resolution X-ray semiconductors scans. We illustrate our approach to locating the region of interest of the structures, their individual components, and their void defects. We showcase how semi-supervised learning is utilized to capitalize on the vast amounts of available unlabeled data to enhance both detection and segmentation performance. Additionally, we explore the benefit of contrastive learning in the data pre-selection step for our detection model and multi-scale Mean Teacher training paradigm in 3D semantic segmentation to achieve better performance compared with the state of the art. Our extensive experiments have shown that our method achieves competitive performance and is able to outperform by up to 16% on object detection and 7.8% on semantic segmentation. Additionally, our automated metrology package shows a mean error of less than 2 μm for key features such as Bond Line Thickness and pad misalignment.

## 1. Introduction

Quality control and evaluation play a critical part in the semiconductor packaging domain. It is essential to make sure that the fabricated wafers and semiconductor packages have been manufactured as expected and do not include any major defects. Hidden defects in miniaturized interconnects within 2.5D-3D High Bandwidth Memory (HBM) packages are a primary source of low yield. Identifying these defects is both challenging and time consuming. The prevalent failure analysis approach involves a destructive process of cross-sectioning a semiconductor package. Following this, optical inspection is used to detect embedded process defects such as solder extrusions, pad misalignment, embedded voids, and solder shorts. Typically, this failure analysis step is performed manually, which is aided by standard image processing techniques. However, this method demands significant effort, man-hours, domain expertise, and costly tools. Moreover, it only provides information on a single 2D plane, necessitating repetition if more information is needed in adjacent regions. Modern 3D X-ray machines can offer a satisfactory resolution for examining and analyzing concealed features such as Through Silicon Vias (TSVs), micro-bumps, and other metallic structures. This method could serve as an exceptional non-destructive failure analysis technique in the future. At present, scanning takes 2–8 h per sample, and some areas are affected by glaring artifacts such as beam hardening [1]. As a result, these tools may not be practical for real-world deployment. However, like any technology, future advancements will help decrease acquisition time and enhance the quality of these scans.

Artificial Intelligence (AI) has significantly impacted technological advancements in various fields, such as visual surveillance, predictive maintenance, object detection, and image segmentation. Specifically, due to computing advancements and a growing focus on efficient machine learning methods, the scientific community has made great strides in the 3D deep learning domain. Recently, deep learning has been utilized for 2D–3D detection and segmentation tasks in buried packages [2]. Usually, a substantial amount of labeled data is needed to train an accurate model using deep learning-based approaches. This process demands numerous man-hours and can be extremely costly. In a fast-paced world where a chip may undergo multiple revisions annually, companies may not have the time or resources to spend months developing deep learning models for failure analysis that might become obsolete within a year. We build upon our previous work, which developed fully supervised learning (FSL)-based 2D object detection and segmentation models, which is followed by a 2D semi-supervised segmentation learning (SSL)-based approach. We introduce a novel hierarchical consistency regularized Mean Teacher framework for performing 3D object detection and segmentation on 3D X-ray scans, consisting of dense High Bandwidth Memory (HBM) packages with only a limited amount of 3D labeled data. This method employs an efficient and advanced AI-based automated attribute measurement technique that delivers crucial information about the HBMs, such as Bond Line Thickness (BLT), solder extrusion, void-to-solder ratio, and pad misalignment. As labeled and diverse semiconductors data are usually very difficult and expensive to obtain, a semi-supervised approach delivers better detection and segmentation accuracy with less labeled data.

**Figure 1:**
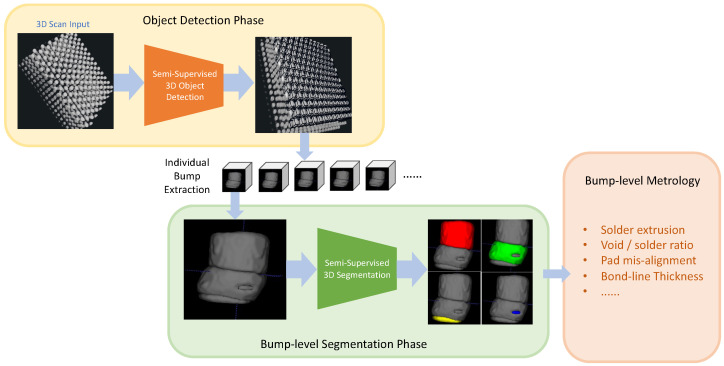
Our proposed method to perform accurate 3D metrology on 2.5D-3D bumps. First, we locate each bump individually in the 3D scan using our multi-view semi-supervised object detection. Second, we employ our 3D semi-supervised segmentation model to identify each component of the bump. Finally, we use our custom 3D metrology Python toolbox to measure and identify various defects such as pad misalignment, void-to-solder ratio, Bond Line Thickness, etc.

In this paper, we describe our innovative approach for identifying HBMs using multiple views and contrastive learning. We further extend this approach by employing semi-supervised object detection to enhance performance. After locating these bumps in 3D X-ray Machine (XRM) data, we isolate them into individual Regions of Interest (RoI). In the second step, these RoIs are processed by another novel semi-supervised based segmentation model capable of identifying various components such as Copper Pillar (CuPillar), Copper Pad (CuPad), Solder, and Void defects. Figure 1 presents our overall approach from 3D scans to metrology analysis. Our contributions are summarized as follows:

- A multi-view SSL 2.5D object detection approach to accurately identify each HBM bump using contrastive learning as data pre-selection.
- An improved multi-scale 3D SSL semantic segmentation method for recognizing individual components of HBMs as well as void defects.
- A 3D Metrology package that performs data cleaning and measures critical features relevant for HBM failure analysis.

We present the related works in Section 2. We introduce our proposed method in Section 3. A detailed description of our multi-view semi-supervised object detection approach is in Section 3.1, our 3D semi-supervised image segmentation methodology is in Section 3.2 and our 3D metrology is in Section 3.3. Section 4 investigates our object detection and 3D segmentation approach, showcasing our capabilities by displaying end results. In particular, Section 4.4 demonstrates our result on 3D metrology. Lastly, we conclude this work in Section 5 and discuss the gaps in the current approach and potential future directions.

## 2. Related Work

### 2.1. Object Detection

Object detection has been extensively studied in computer vision over the years and applied to many applications in autonomous navigation [3], medical imaging [4], and robotics [5]. The advances in deep learning significantly improved the results thanks to higher computational capabilities, huge public datasets, and new detector architectures. There are two main categories of object detectors: one-stage detectors and two-stage detectors. One-stage detectors include YOLO [6,7] and SSD [8]. Those models directly detect the objects on the grid after the feature representation. They usually have faster inference and require less computational load. The two-stage detectors include faster-RCNN and its derivatives [9]. They have an additional region proposal network as an intermediate step. They usually have better accuracy but require more computational load. Object detection has also been applied to 3D scans in medical imaging [10] and semiconductors [2,11]. It is often included as a first step in specific frameworks, as the size of 3D scans is often too big to be processed as a whole. Object detection can then select and extract the regions of interest for further processing. The usual applications are quality control, defect detection, and metrology.

Existing object detector models still require a huge amount of annotated data in order to achieve good performance. Current detectors can take advantage of big public datasets such as MS COCO [12]. However, in semiconductor applications, annotations require expert domain knowledge, and generating relevant data is time-consuming. Thus, it is then difficult to train an object detector to achieve an accurate performance. Semi-supervised learning [13,14] has been introduced to tackle this issue. This approach leverages a small portion of labeled data and unlabeled data and is able to have a better performance compared to fully supervised methods [15,16]. Their architecture is also different and they rely on data augmentations, mutual learning, and pseudo-labels. Semi-supervised frameworks such as Mean Teacher [17] and Unbiased Teacher [18] were published in the literature and outperformed fully supervised methods in the object detection task. However, their choice of unlabeled data was not optimal. It actually relied on a random selection among the dataset and according to the split between labeled and unlabeled data. Depending on the data distribution of data in the datasets, the random choice may introduce a bias in the detection results, and the labeled data may not include a representative sampling of the dataset, which limits the accuracy of the detection.

Contrastive learning has been introduced to learn features that are common and uncommon between classes. MoCo [19] and simCLR [20] demonstrated promising results on unsupervised training representations. Unsupervised learning generally involves two aspects: loss functions and pretext tasks [19]. Contrastive or adversarial losses have been widely used for unsupervised learning. Contrastive losses [21] focus on the similarity between sampling pairs, while adversarial losses [22,23] focus on the difference between probability distributions.

### 2.2. Semantic Segmentation

The field of semantic segmentation using deep learning methods has been widely studied and applied to various domains, including medical imaging [24] and semiconductor materials [1]. Many scenarios require dense mask predictions to reveal and identify the internal structures present in 3D regions.

The advent of deep learning techniques, particularly convolutional neural networks (CNNs), has led to significant advancements in semantic segmentation tasks. Early works in the field employed fully convolutional networks (FCNs) to perform an end-to-end pixel-wise classification of input images [25]. Following the success of FCNs, various network architectures have been proposed to improve the performance of semantic segmentation. Some notable examples include the U-Net [26], which introduced skip connections between the encoding and decoding paths to improve the localization of segmented objects, and the V-Net [27], a 3D extension of the U-Net architecture specifically designed for volumetric data. Annotation difficulty, data complexity, and class imbalance [28] are some of the major challenges in 3D segmentation. Some recent improvements have introduced strong data augmentations for better generalization ability [29] and adopted various loss functions [30,31].

The scarcity of labeled data and the expensive annotation process in many application domains have motivated the exploration of semi-supervised learning techniques for semantic segmentation. Semi-supervised learning aims to improve model performance by leveraging a large amount of unlabeled data alongside a smaller labeled dataset. Various approaches have been proposed for semi-supervised semantic segmentation, such as adversarial training [32] and self-training [33]. These methods share the common goal of leveraging the information in the unlabeled data to enhance the learning process and improve model performance. The recent success of semi-supervised learning emerges under various tasks involving the teacher–student training paradigm. Several self-ensembling methods, such as Mean Teacher [17], are introduced as a consistency regularization method to counter different perturbations between the student and the teacher model.

Following the spirit of Mean Teacher, many achievements have been made to further improve teacher–student training, such as enhanced shape-awareness [34]. In addition, various consistency-based methods are proposed to improve the semi-supervised performance, including uncertainty-aware consistency [35], transformation consistency [36], multi-task consistency [37], and multi-scale consistency [38]. Recent studies indicate that multi-scale consistency is a straightforward yet effective approach for enforcing consistency between different networks at various scales, achieving great success in many tasks [39,40]. Moreover, the feature maps of hidden layers in networks can be extracted to produce multi-scale predictions for deep supervision, improving the discrimination capability.

In this work, we build upon the existing literature by employing a semi-supervised Mean Teacher method with multi-scale V-Net pyramid architecture for the semantic segmentation of 3D semiconductor memory and logic bump data. Our approach aims to leverage the strengths of deep learning-based semantic segmentation, the Mean Teacher paradigm, and semi-supervised learning techniques to address the challenges associated with the limited availability of labeled data in this domain.

## 3. Our Approach

Each XRM scan includes 1000 slices of a resolution 1000×1000. Due to memory limitations and efficiency, processing complete scans consisting of 1 billion voxels increases the processing time and hardware requirements. As we are only interested in individual memory and logic bumps, it is also not efficient to do so since the regions of interest are limited. We introduce a multi-step framework that includes object detection to detect and extract memory and logic bumps and image segmentation to identify the defects and core components such as Copper Pads and Copper Pillars (Figure 1). In our slice-and-fuse approach for object detection, we first process each slice individually rescaled to an input size of 640×640.

### 3.1. Object Detection

**Figure 2:**
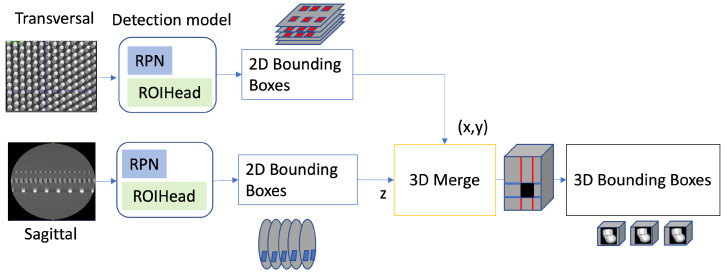
Three-dimensional (3D)-slice-and-fuse approach with the detection model. Both detectors will run on transversal and sagittal views and output 3D bounding boxes corresponding to the bumps in the scans.

Object detection is the first step in our approach. The objective is extract each individual memory and logic bump for the 3D scans. As labeled data are scarce and difficult to obtain, we use a semi-supervised learning approach to reduce the amount of labeled data required and a contrastive learning method to select the most informative labeled data for a better training. Figure 2 shows the overview of the method. We select Detectron2 [41] as our backbone detector in our semi-supervised learning framework as it demonstrated the best accuracy over other detectors [42,43]. The results have been published in [18] over PASCAL VOC [44] and MS COCO [12] datasets.

**Figure 3:**
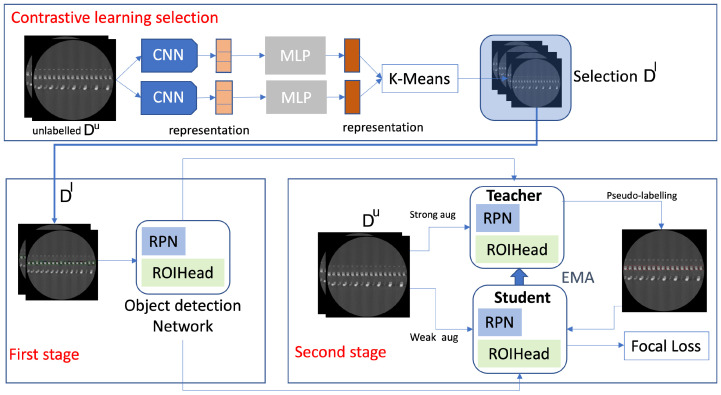
Semi-supervised object detection training with contrastive learning selection. The most informative images are first selected using a simCLR model with k-means clustering. Semi-supervised learning object detection includes a first stage (burn-in) with labeled data and a second stage with unlabeled data and a student–teacher mutual learning framework.

Figure 3 presents the semi-supervised learning approach for detection. We selected Unbiased Teacher [18] as our baseline semi-supervised learning framework and we apply the simCLR [20] method for unsupervised feature extraction. We first define two sets of labeled $D^{l}$ and unlabeled $D^{u}$ data for memory and logic bumps. The splits between $D^{l}$ and $D^{u}$ range from 1% to 10% of our complete dataset. For each 3D sample of the dataset, we select their slices $I_{i=0,...,n}^{s,t}$ from the sagittal and transversal views. The objective is to use as little labeled data as possible for better efficiency and productivity. Unlike other semi-supervised learning methods, we use contrastive learning to select the most informative images in our dataset to reduce data distribution bias rather than using random sampling. The simCLR model [20] is used for feature representation on 2D images $I_{i}^{s,t}$. The method does not use any prior information about the dataset. Different data augmentations are applied to the images such as resizing, noising or blurring. Then, the images are passed in a Resnet50 [43] to generate the feature representation of the images. During the training phase, the feature vectors are passed to a projection head. This projection head includes a MLP with a hidden layer and is used to further refine the feature representation of the images. The objective of the training is to minimize the distance between images containing the same object and maximize the distance between images that include different objects. Once the training is complete, the projection head is discarded, and the feature vectors h(i) are directly obtained as outputs of the encoder (Resnet50). After the feature vector generation, we use a k-means clustering method to select the most appropriate images. Given a set of *k* features {h(i)}, the goal is to partition them into *n* clusters $C=C_{1},C_{2},...,C_{n}$, where the intra-cluster variance is minimized. The objective is to find:

(1) $$argmin\sum_{j=1}^{k}\sum_{h(i)}{\left\|\left(i\right)-\mu_{j}\right\|}^{2}=argmin\sum_{j-1}^{k}|C_{j}|Var\left(C_{j}\right)$$

where $\mu_{j}$ is the mean of feature points in $C_{j}$.

Once the visual features are computed, we use the k-means clustering method where each cluster represents a group of similar images. The number of clusters corresponds to the number of images to be included in $D^{l}$ as we select one image per cluster for annotation. The remaining images will then be stored in $D^{u}$. Figure 3 shows the training process with the supervised learning framework with our data pre-selection.

Once $D^{l}$ and $D^{u}$ are defined, we train our semi-supervised object detection model. It consists of two stages. The first stage, the burn-in stage, initialized the model with $D^{l}$. This model is then duplicated to student and teacher models. The training process follows a mutual-learning framework where the teacher outputs pseudo-labels on unlabeled images and the student updates the teacher’s weights through the Exponential Moving Average (EMA). We also integrate focal loss as it outperforms the original cross-entropy loss due to biased data. The output of the object detection model is a set of 3D bounding boxes defined by [x,y,z,w,h,d] corresponding to the location and dimensions of the bumps for each scan. We previously introduced a slice-and-fuse approach for object detection [11]. The main idea is to run a 2D detector on each sagittal slice $I_{i}^{s}$ which outputs 2D bounding boxes ${\left[x,y\right]}_{n}$ for each bump *n* and then concatenate the results into 3D bounding boxes ${\left[x,y,z,w,h,d\right]}_{n}$. In order to improve the robustness of the detector on defectives bumps, we used a 3D slice-and-fuse approach where we process both sagittal and transversal views instead of a single one. This limits the ambiguity that may arise when the 2D shape of the bumps cannot be separated from each other due to the defects. When slices from two views are used, the ambiguity can be resolved because it does not appear on the second view. Therefore, the concatenation of the 2D slices into 3D is performed. The sagittal view contributes to the x,y directions of the 3D bounding, and the transversal view contributes to the *z* direction.

### 3.2. Semantic Segmentation

After the extraction of individual die structures, semantic segmentation is applied at the bump level to differentiate the volumetric structure and thus identify manufacturing defects in the 3D metrology step. The normal structure of memory and logic bump consists of four foreground components as regions of interest: Copper Pillar (Cu-Pillar), Solder, Copper Pad (Cu-Pad), and Void. Each of the input bumps has a dimension of 100×100×100 approximately. This resolution corresponds to the size of each individual bump after extraction by the object detection phase. It also affects the 3D scanning resolution and the actual size of the bumps (in nm). In ideal cases, each bump should have the same size leading to the same 3D resolution on the scans. However, since our bumps are affected differently by defects and selected fabrication parameters, their size is not identical. By accurately depicting internal volumetric structures, we can facilitate further study on 3D metrology.

The Mean Teacher paradigm, first introduced by Tarvainen and Valpola [17], is a consistency regularization method for semi-supervised learning. This approach involves training two models in parallel: a student model and a teacher model. The student model learns from the labeled dataset and generates predictions on the unlabeled dataset, which can then be used as pseudo-labels for the teacher model in some tasks. We observe that the model tends to overlook the topological relation of semantic components and ignore the wider contextual information. Following our prior work [38,39,40], we adopt additional prediction layers to supervise the quality of hierarchical hidden representations. Deep supervision serves as a minimizer for multi-level segmentation loss, and it is a powerful optimizer to regularize hierarchical consistency and maximize the knowledge learned from unlabeled data.

**Figure 4:**
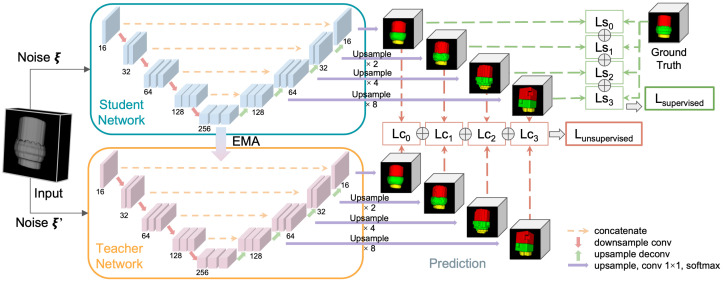
Multi-scale Mean Teacher architecture.

In this work, we select 3D V-Net [27] as the backbone model. To exploit the hidden representation, several auxiliary layers are included after each block of the decoding stage to form a hierarchical feature group. Given that the V-Net structure consists of a downsampling encoder and an upsampling decoder each having multiple stages which preserve the feature information in latent space during the early stages of upsampling, we can assemble the hidden features systematically. In particular, we derive the structure from prior work [38]: each auxiliary layer consists of an upsampling layer, a single channel convolution, and a softmax layer. For each labeled sample, we aggregate the loss between predictions at all scales and ground truth for deeply supervised regularization. Figure 4 illustrates the architecture of our solution. By leveraging multi-scale predictions for deep supervision and consistency regularization, we have stronger control during the training process. Specifically, we encourage consistency between the outputs from different levels of the teacher and student models for the given unlabeled data while also using supervised losses at multiple scales for learning from labeled data. The approach has shown promising results in experiments on various datasets and tasks [38,39,40].

### 3.3. Three-Dimensional (3D) Metrology

**Figure 5:**
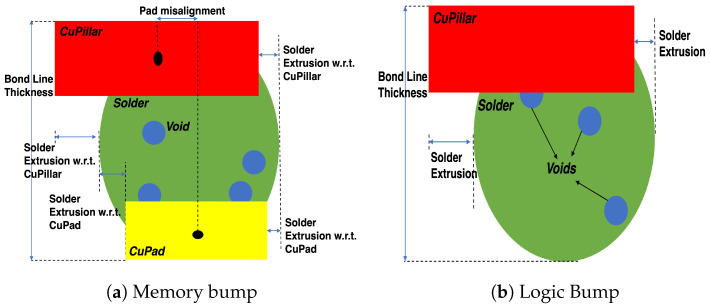
Our 3D metrology features for HBM bumps. The features are computed in 3D using cross-sectional results. We only show a 2D slice illustration for ease of understanding the metrology approach. BLT for logic only includes the vertical height of the Solder and Copper Pillar components.

We develop a custom 3D metrology module to measure critical features that are important for failure analysis of 3D HBM bumps. In particular, we measure the Bond Line Thickness (BLT), solder-to-void-ratio, pad misalignment, and solder extrusion. These features are shown in Figure 5.

Once we receive the predicted output from our multi-scale Mean Teacher 3D segmentation model, we carry out a number of post-processing procedures. Firstly, we ensure that the bumps are aligned vertically, ensuring that they are upright in a shared view. Secondly, we utilize morphological functions such as dilation and erosion to confirm that any pixels within the Solder, Copper Pillar, or Copper Pad components that were classified as background are accurately labeled as the relevant component. Thirdly, we superimpose the predicted voids on top of the newly refined predictions. Finally, we maintain all the forecasted voxels for each category that falls within a specific threshold to the center of mass (CoM) of each category, thereby eliminating any remaining clusters or neighboring component predictions that may be observed in the cropped individual 3D bumps.

We establish the characteristics needed to conduct our metrology, mainly the CoM, top, left, right, and bottom-most areas for each specific bump following the post-processing stage. The characteristics, as demonstrated in Figure 5, are computed for every bump and shared with domain experts to make crucial decisions regarding HBM failure analysis.

## 4. Experiments

### 4.1. Data Fabrication

**Figure 6:**
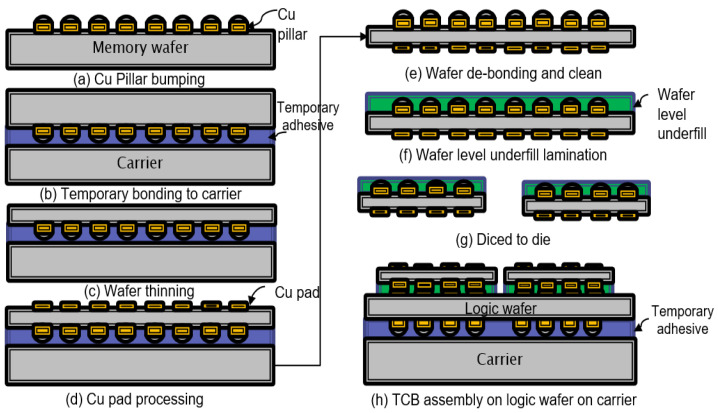
Fabrication steps for HBM bumps [1].

Our dataset includes fabricated 2.5D test vehicles (TV) that resemble contemporary High-Performance Computing packages, in particular, logic and memory bumps. Daisy-chain silicon chips are produced to represent the DRAM and Logic dies which are assembled on top of each other to form High Bandwidth Memory (HBM) cubes. To increase the diversity of the data, we purposely use sub-optimal parameters during the packaging phase to induce defects. For more details on memory and logic bumps fabrication, the readers can refer to [45]. Figure 6 shows the fabrication of HBMs bumps.

Following the fabrication process, we create our 3D scans utilizing a 3D X-ray microscopy (XRM) scanner [46]. The benefit of employing a 3D XRM lies in its ability to facilitate Non-Destructive Techniques (NDTs) for inspecting the fabricated data in various quality assessments. Test vehicles are mounted on sample holders and placed on the XRM autoloader. Subsequently, they are rotated incrementally from −3${}^{\circ }$ to 183${}^{\circ }$ to acquire raw 2D X-ray scans. These 2D scans, combined with geometric information, are processed by a proprietary algorithm to generate the 3D X-ray scans computationally. This computed tomography procedure enables the visualization of chips in 3D, where hidden structures can be imaged at high resolution. Each 3D scan’s resolution is approximately 1000×1000×1000 in size, i.e., 1 billion voxels. One 3D scan represent one TV with a specific set of parameters. The different components of each memory and logic bumps are then labeled manually as Copper Pillar, Copper Pad, Solder and Void. This labeling step has been completed by our annotation team together with the semiconductors fabrication experts. Their knowledge helped the annotation process as they set the fabrication parameters and knew what the desired output is. For ambiguous cases where the boundaries between each class were not obvious, they helped to define and validate the annotations.

### 4.2. Object Detection

For object detection, 3D scans are then divided into 12,849, 4486, and 4593 slices is for training, validation, and testing. As mentioned in the previous section, each 3D scan has 1000 slices. As shown in Figure 1, the logic and memory bumps are not present on all the slices; therefore, slices only containing background have been discarded. Our workstation for this work includes an Intel i9-10900X CPU processor with an NVIDIA TITAN RTX 24 GB GPU containing 4608 cuda cores.

**Figure 7:**
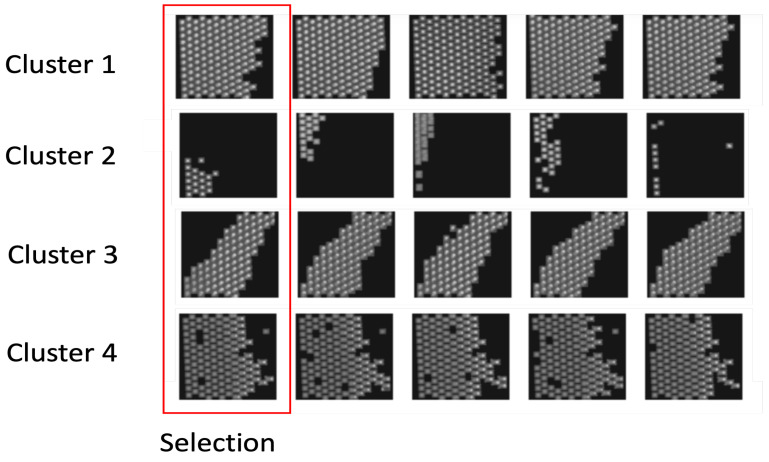
Results of image pre-selection from the contrastive learning method. The first image on the left shows the selection and the images in the same row show similar images assigned to the same cluster. We notice that the selected images are all different and represent different data features in the dataset.

We split our dataset into different amounts of labeled data from 1% to 10% for our experiments. We trained two models for logic and memory, respectively. In our semi-supervised approach Figure 3, we define the weak data augmentation method on the student model as random horizontal flip and the strong augmentation methods on the teacher model as adding color jittering, grayscale, Gaussian blur, and cutout patches. Mean Average Precision (mAP) is used as our evaluation metric, which consists of an Intersection-over-Union (IoU) calculation estimating the quality of the predicted bounding boxes compared to the ground truth data [1] on different thresholds from 0.5 to 0.95. The recall rates of the method are also reported to evaluate the escapes or False Negatives. We first use the simCLR network with pre-trained weights on MS COCO [12] to select the most informative images according to each data split. Figure 7 shows the images per cluster for a split of 1% which corresponds to a selection of four images for training. The training parameters of the semi-supervised model are as follows. The learning rate is 0.01, the initial number of steps (burn-in stage) is 2000, and the total number of steps is 10,000. The EMA rate is 0.996.

As preliminary experiments, we compared the performance of a data selection strategy on a dedicated object detection dataset [5] and against other generic methods and different splits of labeled data. Results are shown in Table 1. We can notice that the best detection results on the mAP metric are obtained with our simCLR approach.

We evaluate the efficacy of the 2D semi-supervised object detection on the individual slices on both sagittal and transversal views with contrastive learning selection. We compare our approach with the baseline (Unbiased Teacher [18]) as well as the fully supervised model (Detectron2 [41]). The baseline includes the first and second stages of the detection approach with a random pre-selection of data. The first stage or “burn-in” stage uses the labeled data to train a detector model. This model is then duplicated into student and teacher models and further trained with the remaining unlabeled data. The fully supervised model Detectron2 includes a first-stage training with labeled data.

Table 2 shows the results for the logic and memory bumps. We observe that our proposed model outperforms the FSL model by up to 10% mAP for logic bumps and up to 16% for memory bumps. We also note that the performance improves for higher splits when more labeled data are used. Our data selection strategy is able to significantly improve the overall detection accuracy. This shows that selected images are representative of the overall dataset and the data distribution bias is limited. The model has received informative images which led to better overall accuracy. On the other hand, the baseline model has a lower accuracy due to fewer informative images used in the first stage. The selected images do not reflect the overall dataset; therefore, the overall detection accuracy is lower.

**Figure 8:**
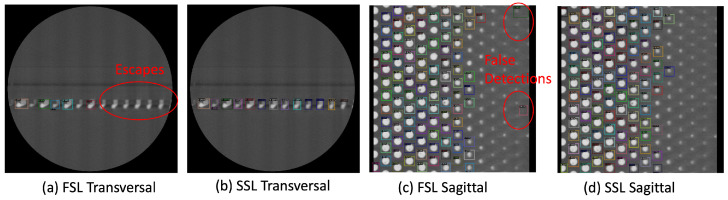
Detection results with fully supervised (Detectron2) FSL (**a**,**c**) and semi-supervised UBT (**b**,**d**) models on logic bumps (2% split). Our approach shows a better accuracy with significantly lower spaces and false detections.

Given the specificity of the data and the low distribution, we notice that our SSL network performs well even with a very small amount of labeled data (1%). This demonstrates the reduced requirement for labeled data with our SSL framework. We also show the detection results on some slices for both sagittal and transversal views to highlight these differences in Figure 8. We observe more false detections on the FSL model compared to ours. The full implementation of the method includes three phases: the first phase detects the bumps on the individual slices, the second estimates the 3D bounding boxes in the scan, and finally, the third phase extracts the bump into individual files. Given the structure of the data and our processing scripts, all phases can be parallelized to reduce the processing time. Furthermore, our method with 1% labeled data has a better detection accuracy than other methods with 10% labeled data for both sagittal and transversal views for logic and memory bumps except for the 10% transversal split for the unbiased teacher.

**Figure 9:**
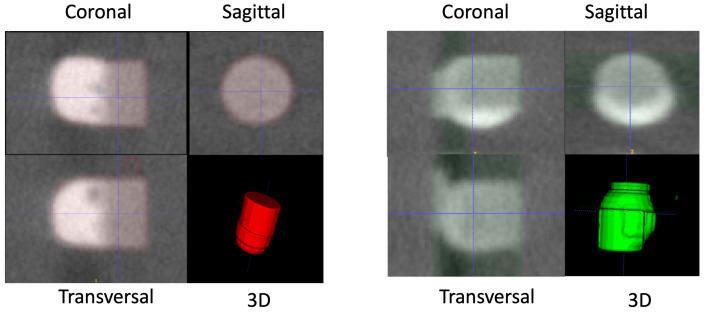
Examples of logic (**left**) and memory (**right**) bumps extracted from the 3D scans. Three views (transversal, sagittal, coronal) are shown with the 3D representation.

The results show that a semi-supervised learning approach with data selection is able to provide better detection accuracy with less labeled data available. As semiconductor data are difficult and expensive to obtain, our approach is able to leverage limited labeled data and use unlabeled data to perform the bump extraction task. Our method was able to outperform the baseline and fully supervised model on all splits from 1% to 10% of labeled data. Our contrastive learning selection demonstrated an improvement of up to 9% on the mAP accuracy over the baseline for both memory and logic bumps and up to 16% over the fully supervised method. Given the structured data in HBMs, our method shows that labeled data on the full dataset are now not required, and a semi-supervised method with a fraction of labeled data is able to perform the extraction task. Our slice-and-fuse approach shows that processing 2D slices with a concatenation can leverage on the good accuracy of 2D detectors and reduce the memory requirement of high-resolution 3D scans. Figure 9 shows the detection and cropping of logic and memory bumps from the 3D scans in each view and their 3D rendering.

### 4.3. Semantic Segmentation

For our segmentation experiment, we have in total {76, 36} bump-level training 3D and {13, 7} testing scans for memory and logic data. Subsets of 2.5%, 5%, 10%, 50%, and 100% labeled data are employed for training. The hardware used is identical to that in Section 4.2.

We establish our comparison between three different setups: fully supervised run using V-Net backbone, naive Mean Teacher semi-supervised run using the same V-Net structure, and the proposed semi-supervised multi-scale Mean Teacher method using V-Net with auxiliary layers. By comparing each training mode under various data percentages, we are able to concretely evaluate the effectiveness of the proposed method.

Similar to our object detection experiments, we adopt different optimization metrics for supervised and unsupervised parts training. The supervised loss is computed using the multi-class Dice loss function, which measures the similarity between the predicted segmentation maps and the ground truth labels. The consistency loss is calculated as the Mean Squared Error (MSE) between the student and teacher models’ predictions on the unlabeled dataset. The overall loss function is defined as the weighted sum of the supervised loss and the consistency loss. Both the supervised loss on the labeled dataset and the consistency loss on the unlabeled dataset from the V-Net pyramid consist of multiple auxiliary losses. We empirically assign scale-wise components with weights 0.5, 0.2, 0.2, and 0.1 for memory runs and 0.6, 0.25, 0.1, and 0.05 for logic runs.

The V-Net model with auxiliary layers is trained with an initial learning rate of 0.01 and step-down decay interval of every 5000 iterations at the scale of 0.1. We adopt a linear learning rate warm-up of 300 iterations and train the backbone network from scratch. The decay parameter of exponential moving average (EMA) update rate is α=0.999, and the consistency weight is set to γ=0.01. When the training initiates, the model experiences a linear consistency ramping-up stage sustained for 40 epochs until full scale. For all modes of experiments in our work, the training lasts for 10,000 iterations using SGD optimizer. Specifically for semi-supervised runs, we preserve the initial 2000 iterations supervised, i.e., our semi-supervised runs consist of 2000 burn-in iterations and 8000 semi-supervised iterations.

**Figure 10:**
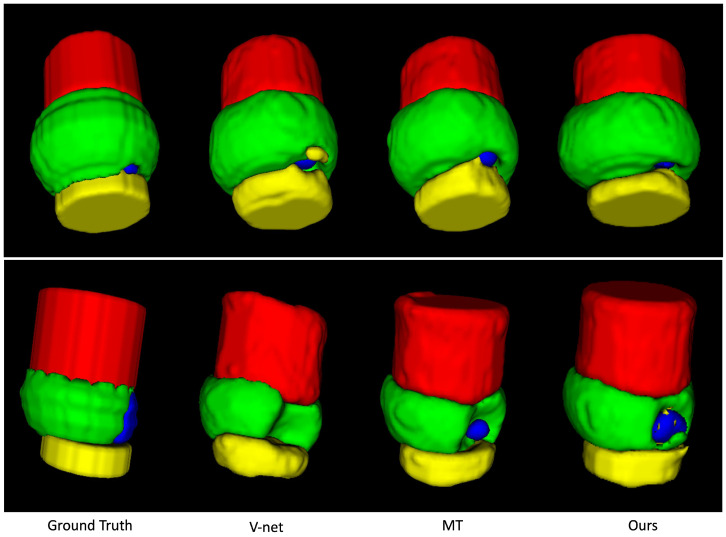
We show one inferred test sample from ground truth annotations, V-Net, Mean Teacher (MT), and our multi-scale Mean Teacher output. Our approach provides visually more consistent and less erroneous results than our baseline.

**Figure 11:**
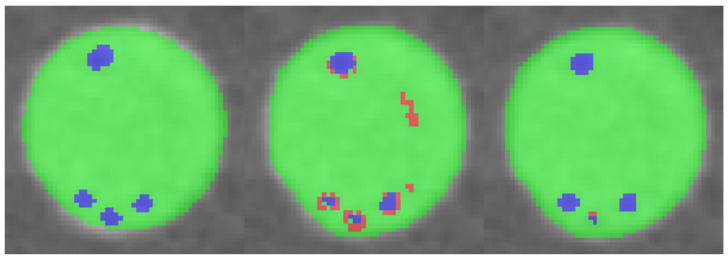
A top–down perspective comparison between ground truth, baseline Mean Teacher and multi-scale Mean Teacher inference results. Our regularized method provides a better control over training process and suppresses overall misclassification.

We evaluate our model performance using multiple quantitative metrics: multi-class Dice coefficient and Jaccard coefficient (IoU). Table 3 shows the Dice and IoU performance between FSL training, Mean Teacher SSL training, and multi-scale Mean Teacher SSL training under various percentages of selected labeled data. We observe that the overall performance increases along with the addition of labeled data. Qualitatively, our method produces less misclassification and better conserves the overall shape of the material structure. Specifically, we achieve nearly 8% improvement on logic bump data. Figure 10 and Figure 11 visualize some inferred test samples through color-coded images. Although multi-scale runs fail to provide better results on fewer training samples, the performance surpasses its counterparts at a higher percentage of data. Empirically, we observe a similar trend in experiments with too strong regularization, leading models to perform less effectively.

### 4.4. Three-Dimensional (3D) Metrology

We perform 3D metrology measurements as described in Section 3.3. We also perform a post-processing step to clean the inference using computer vision techniques as discussed in the previous section. We report our 3D metrology findings in Table 4. The results reflected in the table are averaged across all splits ranging from 1 to 100% labeled data and the remaining data are used as unlabeled data in the supervised learning setting. We observe that aligning, cleaning, and performing neighborhood clustering drastically improves the results when the inference has some serious flaws such as situations when most of the cropped predicted bumps include false positives for Copper Pillars, Copper Pads, and Solders in addition to neighborhood components at the edges. Our final metrology results show a mean error of less than 1.41 μm for Bond Line Thickness, 2.53 μm for solder extrusion, and 0.91 μm for pad misalignment when compared to the ground truth labeled data.

## 5. Conclusions

In this study, we have introduced an innovative framework for facilitating 3D metrology by utilizing cutting-edge 3D Semi-Supervised Deep Learning techniques for object detection and semantic segmentation. We detailed the process of detecting objects across multiple views and merging the results to enhance bump detection performance. Subsequently, we employed 3D semi-supervised semantic segmentation to identify various components within individual structures, such as Copper Pillars, Copper Pads, Voids, and Solder regions. Furthermore, we improve our semi-supervised semantic segmentation by introducing deep supervision and hierarchical consistent regularization. When incorporated with 3D metrology, this approach holds significant promise for decreasing defect analysis duration and consequently boosting measurement accuracy.

We demonstrated that multi-scaled Mean Teacher is able to provide a superior result on HBMs segmentation. Going forward, we plan to explore suitable augmentations that enhance our semi-supervised segmentation method and incorporate balanced regularization. For object detection, future work includes an integration of active and contrastive learning methods for better data selection. The best features for object detection would then be highlighted instead of informative visual features. Finally, the object detection framework may be applied to other domains such as medical imaging or sensors that provide other 3D voxelized scans.

## Figures and Tables

**Table 1:** Comparison of data selection strategy using the Mean Teacher and our improved semi-supervised approach.

Accuracy on Data Selection Strategies (mAP)			
Labeled dataset	1%	5%	10%
LeastConfidence [47]	61.87	78.9	84.34
MarginSampling [48]	62.67	79.12	84.53
EntropySampling [49]	61.34	79.32	84.92
simCLR [20]	**63.07**	**79.54**	**86.56**

**Table 2:** We report the object detection accuracy (Precision and Recall rates) for Logic and Memory dies. Our SSL approach provides more accurate results than Detectron2 [41] (FSL) and Unbiased Teacher [18] (SSL baseline) approaches on both sagittal and transversal views by up to 10% and 18% mAP with IOU = 0.5:0.95 on logic and memory bumps, respectively.

		1%		2%		5%		10%	
IOU = 0.5:0.95		Prec.	Rec.	Prec.	Rec.	Prec.	Rec.	Prec.	Rec.
Memory									
Sagittal	Det2	0.607	0.662	0.631	0.68	0.634	0.685	0.648	0.685
	UBT	0.769	0.801	0.764	0.81	0.788	0.827	0.798	0.832
	Ours	**0.786**	**0.81**	**0.791**	**0.826**	**0.824**	**0.831**	**0.821**	**0.845**
Transver.	Det2	0.723	0.784	0.76	0.79	0.784	0.824	0.803	0.846
	UBT	0.764	0.812	0.781	0.81	0.798	0.834	0.824	0.853
	Ours	**0.843**	**0.873**	**0.854**	**0.879**	**0.874**	**0.892**	**0.886**	**0.916**
Logic									
Sagittal	Det2	0.776	0.806	0.781	0.814	0.782	0.817	0.809	0.848
	UBT	0.795	0.836	0.801	0.841	0.81	0.845	0.814	0.846
	Ours	**0.848**	**0.873**	**0.889**	**0.917**	**0.906**	**0.927**	**0.917**	**0.943**
Transver.	Det2	0.714	0.753	0.659	0.703	0.679	0.725	0.701	0.739
	UBT	0.788	0.824	0.80	0.821	0.824	0.859	0.843	0.873
	Ours	**0.824**	**0.859**	**0.862**	**0.893**	**0.894**	**0.923**	**0.903**	**0.931**

**Table 3:** We report the V-Net FSL and Mean Teacher SSL 3D semantic segmentation results for Memory and Logic dies. The SSL approach is generally able to identify the segments more accurately. Our proposed multi-scale Mean Teacher (MMT) is showing many advantages at higher percentage data, especially for Logic die.

	2.5%		5%		10%		50%		100%	
	Dice	IoU	Dice	IoU	Dice	IoU	Dice	IoU	Dice	IoU
Memory										
V-Net	79.89	64.86	85.14	**77.16**	81.49	73.57	83.26	75.63	87.89	80.19
MT	**80.63**	**72.38**	**85.50**	70.25	**86.69**	71.75	86.10	**82.03**	88.67	**82.81**
**Ours (MMT)**	75.32	64.93	84.83	76.70	86.46	**78.21**	**87.03**	79.21	**89.49**	82.25
Logic										
V-Net	81.82	75.07	84.51	78.74	84.51	78.85	85.26	79.85	84.34	78.55
MT	**84.22**	**78.54**	84.33	78.58	84.80	79.66	85.65	80.54	83.79	78.27
**Ours (MMT)**	57.27	48.18	**91.13**	**84.86**	**92.29**	**86.86**	**92.58**	**87.41**	**91.59**	**86.06**

**Table 4:** We display the mean error for metrology features such as Bond Line Thickness, solder extrusion, and pad misalignment (in μm) between ground truth, MT, ours, and post-processed predictions. We observe that our multi-scale Mean Teacher approach segments the die more accurately, and our metrology package further improves the results significantly.

Metrology Error	MT	Ours	Post-Processed
Memory Die			
Bond Line Thickness	2.19	1.41	**1.41**
Solder Extrusion	3.30	3.27	**2.53**
Pad Misalignment	2.12	0.91	**0.91**
Void-to-Solder Ratio	0.046	0.046	**0.045**
Logic Die			
Bond Line Thickness	3.57	1.63	**1.45**
Solder Extrusion	1.36	1.00	**0.68**
Void-to-Solder Ratio	1.20	**0.0028**	0.0029

## Data Availability

Data is unavailable due to policy restrictions.
